# Association between advanced lung cancer inflammation index and gallstone prevalence among U.S. adults: A population-based study

**DOI:** 10.1371/journal.pone.0321733

**Published:** 2025-04-15

**Authors:** Chaofeng Gao, Miaoyan Liu, Yuan Sun, Zekun Zhao, Fengxian Wei, Xiaodong Xu

**Affiliations:** 1 Department of General Surgery, Lanzhou University Second Hospital, Lanzhou, China; 2 Xi’an Medical University, Xi’an, China; 3 Department of Cardiovascular Surgery, Xi’an Third Hospital, Xi’an, China; Sichuan University, CHINA

## Abstract

**Introduction:**

Gallstones are a common digestive disorder, with a global prevalence of 10%–15%, posing a significant economic burden on public health. The formation of gallstones is closely associated with inflammation and nutritional status. The Advanced Lung Cancer Inflammation Index (ALI) is a composite measure for assessing inflammation and nutritional status; however, its relationship with gallstone risk remains unclear. This study aims to investigate the association between ALI and gallstone prevalence among U.S. adults.

**Methods:**

This study is based on data from the 2017–2020 National Health and Nutrition Examination Survey (NHANES) and includes 5,826 adults aged 20 years and older. The Advanced Lung Cancer Inflammation Index (ALI) was calculated using body mass index (BMI), serum albumin levels, and the neutrophil-to-lymphocyte ratio (NLR). The prevalence of gallstones was determined through questionnaire surveys. Multivariable logistic regression models were employed to analyze the relationship between ALI and the risk of gallstones. Additionally, trend analysis, smooth curve fitting, and subgroup analyses were conducted.

**Results:**

The study results showed a significant positive correlation between ALI levels and the risk of gallstone disease. After fully adjusting for covariates, each unit increase in lnALI was associated with a 42% increase in the risk of gallstone disease (OR =  1.42, 95% CI: 1.12–1.80). Trend analysis indicated a significant dose-response relationship between ALI and gallstone risk (P for trend <  0.01). Subgroup analysis further revealed that the correlation between ALI and gallstone risk was more pronounced in females, non-diabetic patients, individuals with higher education levels, those with insufficient physical activity, and non-drinkers, with gender showing a significant interaction effect (interaction P <  0.05). Smooth curve fitting further validated the linear relationship between ALI and gallstone risk, and this association was particularly prominent in the female population.

**Conclusions:**

This study demonstrates that ALI is significantly associated with the risk of gallstones, particularly among women. As a simple and readily accessible indicator, ALI may help identify high-risk populations and provide a new clinical tool for the prevention and management of gallstones. Future longitudinal studies should further validate these findings and evaluate the predictive value of ALI across different populations.

## Introduction

Gallstones are a common digestive disorder with a global prevalence of 10%–15%, posing a significant health risk to the population [1]. Surgical intervention remains the cornerstone of gallstone management, with approximately 700,000–1,000,000 cholecystectomies performed annually in the United States alone, imposing a considerable economic burden on healthcare systems worldwide [2]. Gallstones are anatomically classified into two main types: those developing in the gallbladder and those forming in the bile ducts. While the majority of gallstone carriers remain asymptomatic, a subset of patients may present with clinical manifestations including abdominal pain, nausea, and vomiting [3]. However, delayed diagnosis in certain cases can precipitate serious complications such as acute pancreatitis, acute cholangitis, obstructive jaundice, and even gallbladder perforation [4,5]. Furthermore, chronic gallstone disease has been associated with an elevated risk of gallbladder carcinoma [6].Therefore, early assessment of gallstone risk is crucial.

The formation of gallstones is primarily due to dysfunction in the gallbladder or bile secretion. Current evidence indicates that gallstones are associated with various factors, including age, gender, obesity, cardiovascular diseases, microbiota, glucose metabolism, and environmental exposures [7]. Recently, studies have shown a close link between inflammation, oxidative stress, and gallstone formation [8,9]. Elevated levels of circulating inflammatory proteins, such as IL-6, IL-10, IL-12, and IL-13, have been found to increase the risk of gallstones [10]. Additionally, overnutrition is also considered a significant factor in increasing the risk of gallstones, as it affects lipid metabolism and bile cholesterol supersaturation, thereby promoting gallstone formation [11]. Hence, it is essential to explore the role of inflammation and nutritional status in gallstone formation further.

The advanced lung cancer inflammation index (ALI) is a novel composite measure utilized to evaluate inflammation and nutritional status, first proposed by Jafri et al. in 2013, and has since been established as an independent prognostic marker in patients with non-small cell lung cancer [12]. Subsequently, Song et al. compared various nutrition- and inflammation-related indicators and found that ALI significantly outperforms other markers in evaluating the prognosis of lung cancer patients [13]. ALI is calculated based on routine clinical tests, including body mass index (BMI), serum albumin level, and the neutrophil-to-lymphocyte ratio (NLR) [14]. These parameters are easily obtainable and simple to calculate, making ALI a practical and widely applicable risk assessment tool. With further research, ALI has gradually been applied to the prognosis of other types of tumors, particularly digestive system tumors. Studies have shown that ALI is an independent prognostic factor for gastric cancer, colorectal cancer, and liver cancer, with lower ALI levels associated with poorer outcomes [15–17]. Additionally, research has revealed that ALI is closely related to the prognosis of various cardiovascular and cerebrovascular diseases, such as hypertension, heart failure, and stroke [18–20]. It is also significantly linked to the long-term prognosis of patients with diabetes [21]. This growing body of evidence suggests that ALI can serve as a versatile tool for disease management and risk stratification.

Given the crucial role of inflammation and nutritional status in the formation of gallstones, ALI holds potential as a predictive tool in this context. ALI integrates BMI, serum albumin level, and NLR—three key factors that are independently associated with gallstone risk—offering a comprehensive evaluation of the influence of inflammation and nutrition. Although previous studies have explored the effects of inflammation or nutritional status on gallstone formation, none have considered the combined effects of these factors. Thus, the potential connection between ALI and gallstone risk remains unclear. This study aims to evaluate the relationship between ALI and the risk of gallstones by analyzing data collected from the National Health and Nutrition Examination Survey (NHANES) from 2017 to March 2020, providing new clinical perspectives and guidance for the prevention and treatment of gallstones.

## Materials and methods

### Study population

Our study employed a population-based, retrospective, cross-sectional design to analyze secondary data from the National Health and Nutrition Examination Survey (NHANES) database. NHANES is designed to evaluate the health and nutritional status of residents across the United States through a comprehensive, multi-stage sampling strategy, ensuring national representativeness. Participants underwent extensive evaluations, including household interviews and assessments at Mobile Examination Centers (MECs), which included physical measurements, clinical examinations, and laboratory tests.

**Fig 1:**
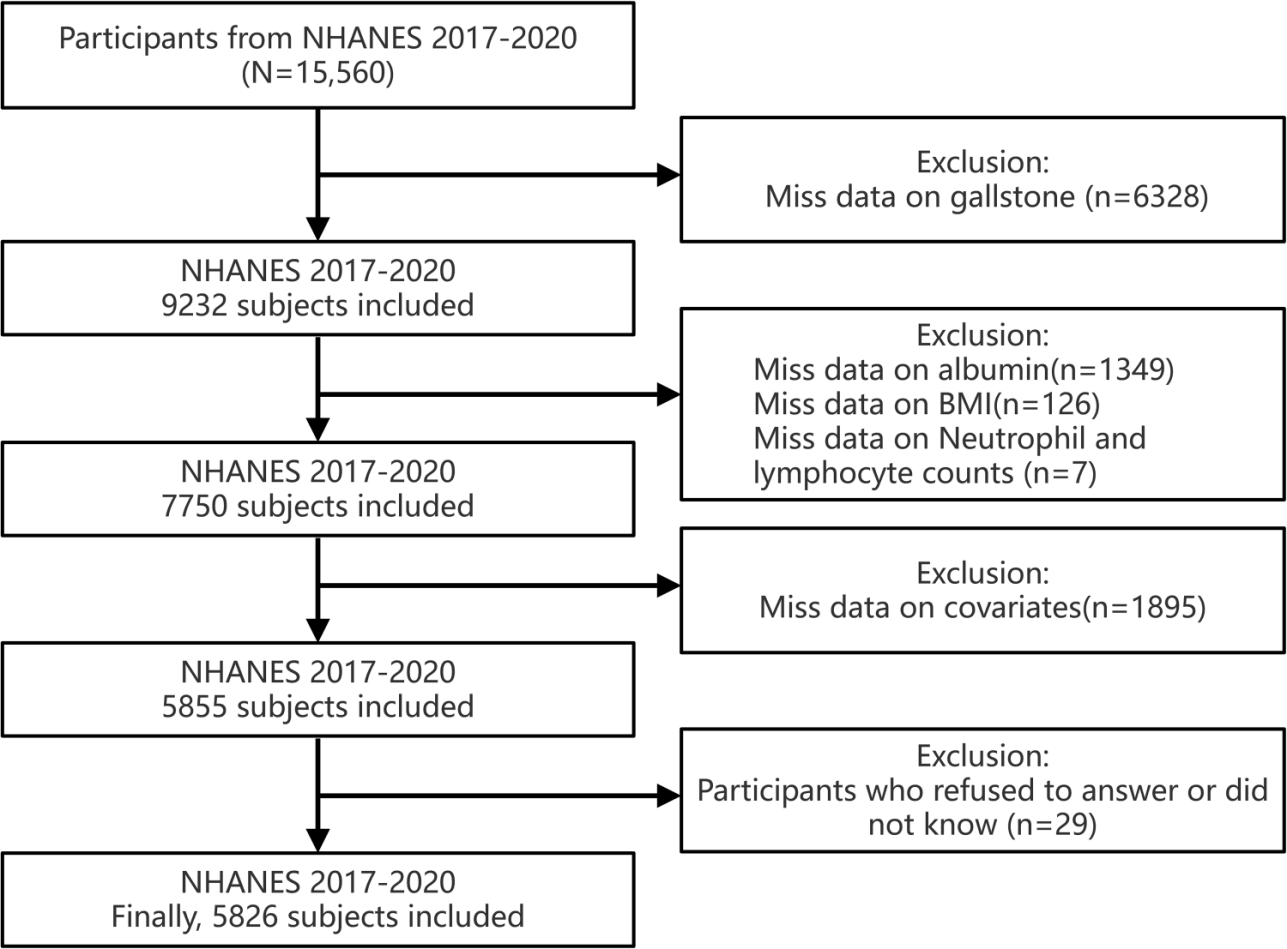
Flowchart of study cohort selection process.

Our study focused on data from the NHANES cycle spanning 2017 to March 2020, as gallstone-related questionnaires were exclusively available during this period. Participants were excluded according to the following criteria: (1) those with incomplete gallstone information; (2) individuals lacking data on albumin, neutrophils, lymphocytes, or BMI; (3) participants with missing covariate data; and (4) those who declined to answer or responded with “don’t know.” Given that the education level covariate was collected only from participants aged 20 and above, this study was restricted to adults aged 20 years or older. The final analysis included a total of 5,826 participants. The detailed screening process is illustrated in Fig 1.

The study protocol of NHANES was approved by the National Center for Health Statistics (NCHS) Research Ethics Review Board, and written informed consent was obtained from all participants. Consequently, no additional ethical approval or participant consent was required for subsequent data analysis.

### Assessment of ALI and gallstone

ALI serves as an indicator of participants’ inflammation and nutritional status, and we utilized it to evaluate its association with gallstone risk. ALI is calculated using the formula: ALI =  BMI (kg/m^2^) ×  albumin level (g/dL)/ NLR, where NLR is derived by dividing the neutrophil count by the lymphocyte count. These parameters were measured at the Mobile Examination Center (MEC). The NHANES Laboratory Manual provides detailed standard methods for assessing serum and plasma markers, discusses potential biases, and offers additional information at the CDC NHANES Biosamples website. Given the skewed distribution of ALI, we applied a logarithmic transformation, using ln(ALI) in our statistical analyses. Gallstone status was determined based on the questionnaire item, “Has a doctor or other health professional ever told you that you have gallstones?” Participants responding “yes” were classified as having gallstones.

### Covariates

Based on clinical expertise and established guidelines, we included the following covariates: age, gender, race, education level, poverty-income ratio (PIR), smoking status, physical activity, alcohol consumption, diabetes, and hypertension. Demographic variables encompassed age, gender, race, education level, and PIR. Health-related behavioral factors included smoking status, physical activity, and alcohol consumption. Chronic disease variables comprised diabetes and hypertension. All data were analyzed using the weighting methodology recommended by NHANES. Education level was categorized as above or below a high school diploma. Smoking status was classified into non-smokers, former smokers, and current smokers, defined as individuals who have smoked fewer than 100 cigarettes in their lifetime, those who have smoked more than 100 cigarettes but are not currently smoking, and those who have smoked more than 100 cigarettes and are currently smoking, respectively. Physical activity was determined based on whether participants engaged in any activity that increased breathing or heart rate during a typical week. Alcohol consumption was defined as consuming any type of alcoholic beverage at least once per month on average over the past year. Diabetes was defined as having an HbA1c level of at least 6.5%, a fasting blood glucose level of 126 mg/dL or higher [22], a self-reported diagnosis by a healthcare professional, or current insulin use. Hypertension was determined by whether a participant had ever been informed by a doctor or other health professional that they had high blood pressure.

### Statistical analysis

In accordance with the guidelines provided on the official NHANES website, a complex sampling design and corresponding sample weights were utilized to generate nationally representative estimates. Continuous variables were expressed as weighted means ±  standard error, while categorical variables were reported as unweighted counts (with weighted proportions). To compare baseline characteristics between participants with and without gallstones, weighted t-tests were employed for continuous variables, and weighted chi-squared tests were used for categorical variables to assess group differences. The relationship between ALI and gallstones was examined using multivariable logistic regression models. Three distinct models were constructed: Model 1 was unadjusted; Model 2 was adjusted for age, gender, and race; and Model 3 was adjusted for all covariates. The results of each model were presented as odds ratios (OR) with 95% confidence intervals (CI). Given the skewed distribution of ALI, a logarithmic transformation (ln(ALI)) was applied. For a more granular analysis, ALI was categorized into quartiles, and trend tests were conducted to evaluate the dose-response relationship between ALI quartiles and gallstone risk. Subgroup analyses were performed to investigate potential effect modifications by gender, diabetes status, age, education level, physical activity, alcohol consumption, and hypertension. Interaction terms between ALI and these variables were incorporated into the regression models to determine their statistical significance. A smoothing curve fitting method was applied to visualize the association between ALI and gallstones, with stratification by gender to assess potential differential effects. Statistical significance was defined as a p-value <  0.05. All data analyses were conducted using R Studio (version 4.3.3) and Empower software (version 4.2).

## Results

### Baseline characteristics

This study ultimately included 5,826 participants, with a mean age of 48 ±  17 years. Among them, 49% were male and 51% were female, with 67% identified as Non-Hispanic White. The mean ln(ALI) value was 4.14 ±  0.49. Participants were stratified into two groups based on the presence of gallstones, with 11% of the cohort diagnosed with gallstones. Significant differences (P <  0.05) were observed between the two groups for age, gender, BMI, physical activity, smoking status, diabetes, poverty-income ratio (PIR), alcohol consumption, and hypertension. Participants with gallstones were more likely to be female, older, have a higher BMI, a history of smoking, diabetes, or hypertension, abstain from alcohol, engage in less physical activity, and have lower economic status (Table 1).

**Table 1:** Study population characteristics.

Characteristic	Gallstone			*P*-value^2^
	Overall, N = 5826 (100%)^1^	No, N = 5206 (89%)^1^	Yes, N = 620 (11%)^1^	
**Age (years)**	48.00 ± 17.00	46.00 ± 16.91	59.00 ± 15.31	**<0.001**
**Gender**				**<0.001**
Male	2938 (49%)	2758 (52%)	180 (26%)	
Female	2888 (51%)	2448 (48%)	440 (74%)	
**Race**				0.056
Mexican American	684 (8.0%)	602 (8.1%)	82 (7.1%)	
Other Hispanic	571 (6.8%)	500 (6.8%)	71 (6.9%)	
Non-Hispanic White	2278 (67%)	1990 (66%)	288 (72%)	
Non-Hispanic Black	1460 (10%)	1350 (10%)	110 (6.1%)	
Other race	833 (8.3%)	764 (8.4%)	69 (7.6%)	
**Ed**uca**tion level**				0.085
≤High school	2318 (36%)	2057 (35%)	261 (41%)	
>High school	3508 (64%)	3149 (65%)	359 (59%)	
**BMI group**				**<0.001**
<25	1412 (25%)	1346 (27%)	66 (11%)	
25–30	1820 (31%)	1650 (31%)	170 (31%)	
≥30	2594 (44%)	2210 (42%)	384 (58%)	
**Physical activity**				**0.026**
Yes	2856 (56%)	2606 (57%)	250 (49%)	
No	2970 (44%)	2600 (43%)	370 (51%)	
**Smoking status**				**<0.001**
Never	3173 (55%)	2872 (56%)	301 (46%)	
Former	1537 (28%)	1328 (27%)	209 (37%)	
Current	1114 (17%)	1005 (17%)	109 (17%)	
**Diabetes**				**<0.001**
Yes	1161 (15%)	960 (13%)	201 (25%)	
No	4665 (85%)	4246 (87%)	419 (75%)	
**PIR**	3.39 ± 1.62	3.41 ± 1.63	2.92 ± 1.55	**0.002**
**Alcohol**				**<0.001**
Yes	3015 (57%)	2772 (59%)	243 (42%)	
No	2811 (43%)	2434 (41%)	377 (58%)	
**Hypertension**				**<0.001**
Yes	2239 (33%)	1911 (31%)	328 (48%)	
No	3587 (67%)	3295 (69%)	292 (52%)	
**ln ALI**	4.14 ± 0.49	4.14 ± 0.49	4.15 ± 0.49	0.2

^1^Median ± SD for continuous; n (unweighted) (%) for categorical.

^2^T-test adapted to complex survey samples; chi-squared test with Rao & Scott’s second-order correction.

### The relationship between ALI and gallstones

To ensure the robustness of our model, we evaluated multicollinearity and correlations among the variables. The calculated Generalized Variance Inflation Factor (GVIF) values (S1 Table in S1 File) were all below 10, indicating no significant multicollinearity. The Spearman correlation heatmap (S1 Fig in S1 File) revealed that most variables exhibited weak correlations (|ρ| <  0.5), suggesting minimal overlap among them. Based on these preliminary assessments, we proceeded to examine the relationship between ALI and gallstones using three multivariable regression models, as presented in Table 2. A positive association was observed between ALI and gallstone presence. In Model 1, which did not adjust for any covariates, the relationship between log-transformed ALI and gallstones was not statistically significant. However, in Model 2, after adjusting for age, gender, and race, a significant correlation emerged between ln ALI and gallstone risk (OR: 1.46, 95% CI: 1.10–1.94). In Model 3, which further adjusted for education level, smoking status, physical activity, alcohol consumption, hypertension, PIR, and diabetes, the association between ln ALI and gallstones remained significant (OR: 1.42, 95% CI: 1.05–1.92). Each one-unit increase in ln ALI was associated with a 42% higher likelihood of developing gallstones. To further explore the complex relationship between ln ALI and gallstone risk, we stratified ln ALI into quartiles for analysis. The results demonstrated that in both Model 2 and Model 3, higher quartiles of ln ALI (particularly Q4) were strongly associated with an increased risk of gallstones (Model 2: OR for Q4: 1.69, 95% CI: 1.14–2.52; Model 3: OR for Q4: 1.61, 95% CI: 1.04–2.50). The trend test revealed a significant linear relationship between ln ALI quartiles and gallstone risk (Model 3: p for trend =  0.032).

**Table 2:** The association between ALI and gallstones.

	Model 1	Model 2	Model 3
	OR (95% CI)	OR (95% CI)	OR (95% CI)
ln ALI	1.14 (0.91, 1.42)	1.46 (1.10, 1.94)	1.42 (1.05, 1.92)
ln ALI			
Q1	Reference	Reference	Reference
Q2	1.01 (0.71, 1.42)	1.12 (0.75, 1.67)	1.14 (0.72, 1.81)
Q3	0.86 (0.63, 1.18)	1.09 (0.77, 1.54)	1.11 (0.77, 1.61)
Q4	1.22 (0.89, 1.68)	1.69 (1.14, 2.52)	1.61 (1.04, 2.50)
*P* for trend	0.306	**0.012**	**0.032**

Model 1: no covariates were adjusted. Model 2: age, gender and race were adjusted. Model 3: age, gender, race, education level, smoking status, physical activity, alcohol, hypertension, PIR and diabetes were adjusted.

### Subgroup analysis and interaction testing

To further evaluate the robustness and heterogeneity of the association between ALI and gallstones, we performed subgroup analyses stratified by gender, diabetes status, age, education level, physical activity, alcohol consumption, and hypertension, with appropriate adjustments for confounding factors. The results revealed that the association between ALI and gallstones was significant in females (OR =  1.70, 95% CI: 1.27–2.27), non-diabetic individuals (OR =  1.42, 95% CI: 1.02–1.97), those with higher education levels (OR =  1.50, 95% CI: 1.09–2.05), individuals with insufficient physical activity (OR =  1.65, 95% CI: 1.16–2.35), and non-drinkers (OR =  1.72, 95% CI: 1.26–2.35). Interaction tests indicated that gender significantly moderated the relationship between ALI and gallstones (P =  0.011), whereas diabetes, age, education level, physical activity, alcohol consumption, and hypertension did not exhibit significant interaction effects on this positive association (Table 3).

**Table 3:** Subgroup analysis between ALI and the prevalence of gallstone.

	Model 1	Model 2	Model 3	*P* for interaction
	OR (95% CI)	OR (95% CI)	OR (95% CI)	
**Gender**				**0.011**
Male	0.64	1.04	1.03	
Female	(0.44,0.92)	(0.64,1.67)	(0.61,1.74)	
**Diabetes**				>0.9
Yes	1.33 (0.88,2.01)	1.38 (0.88,2.15)	1.38 (0.86,2.19)	
No	1.08 (0.84,1.40)	1.42 (1.03,1.96)	1.42 (1.02,1.97)	
**Age**				0.6
<50	0.99 (0.63,1.58)	1.35 (0.81,2.27)	1.23 (0.73,2.09)	
≥50	1.43 (1.07,1.92)	1.37 (0.98,1.92)	1.40 (1.00,1.97)	
**Education**				0.3
≤High school	0.96 (0.69,1.34)	1.33 (0.89,1.98)	1.35 (0.86,2.11)	
>High school	1.30 (0.98,1.71)	1.60 (1.16,2.21)	1.50 (1.09,2.05)	
**Physical Activity**				0.2
Yes	0.97 (0.65,1.44)	1.24 (0.77,1.99)	1.19 (0.73,1.93)	
No	1.36 (1.00,1.85)	1.69 (1.18,2.41)	1.65 (1.16,2.35)	
**Alcohol**				0.3
Yes	0.96 (0.62,1.47)	1.16 (0.71,1.88)	1.14 (0.69,1.90)	
No	1.29 (1.01,1.64)	1.76 (1.31,2.35)	1.72 (1.26,2.35)	
**Hypertension**				0.3
Yes	1.34 (0.92,1.95)	1.60 (1.02,2.50)	1.56 (0.99,2.45)	
No	0.97 (0.73,1.29)	1.27 (0.97,1.66)	1.25 (0.93,1.67)	

Model 1: no covariates were adjusted. Model 2: age, gender and race were adjusted. Model 3: age, gender, race, education level, smoking status, physical activity, alcohol, hypertension, PIR and diabetes were adjusted.

### Smoothed curve fitting

**Fig 2:**
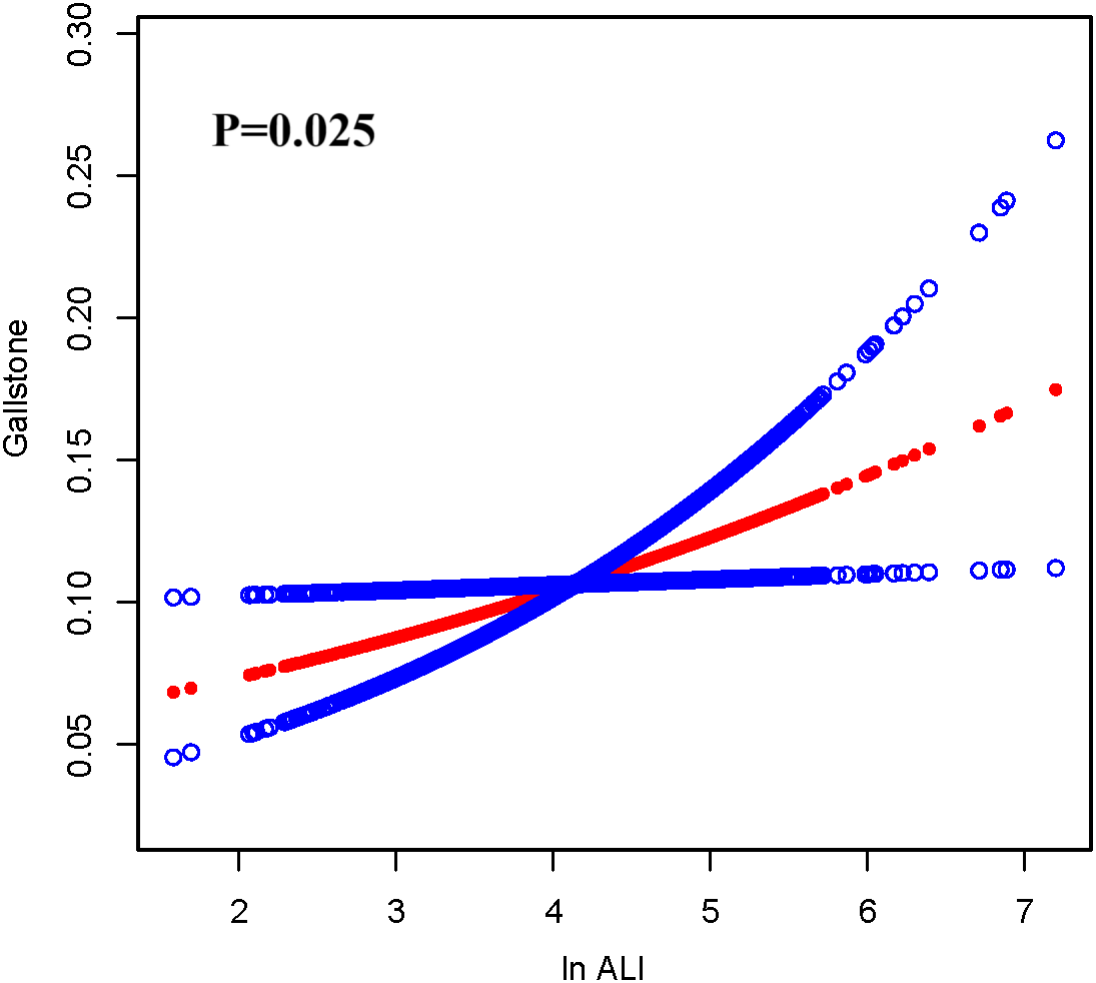
The association between ALI and gallstone. The solid red line represents the smooth curve fit between variables. Blue bands represent the 95% confidence interval from the fit.

**Fig 3:**
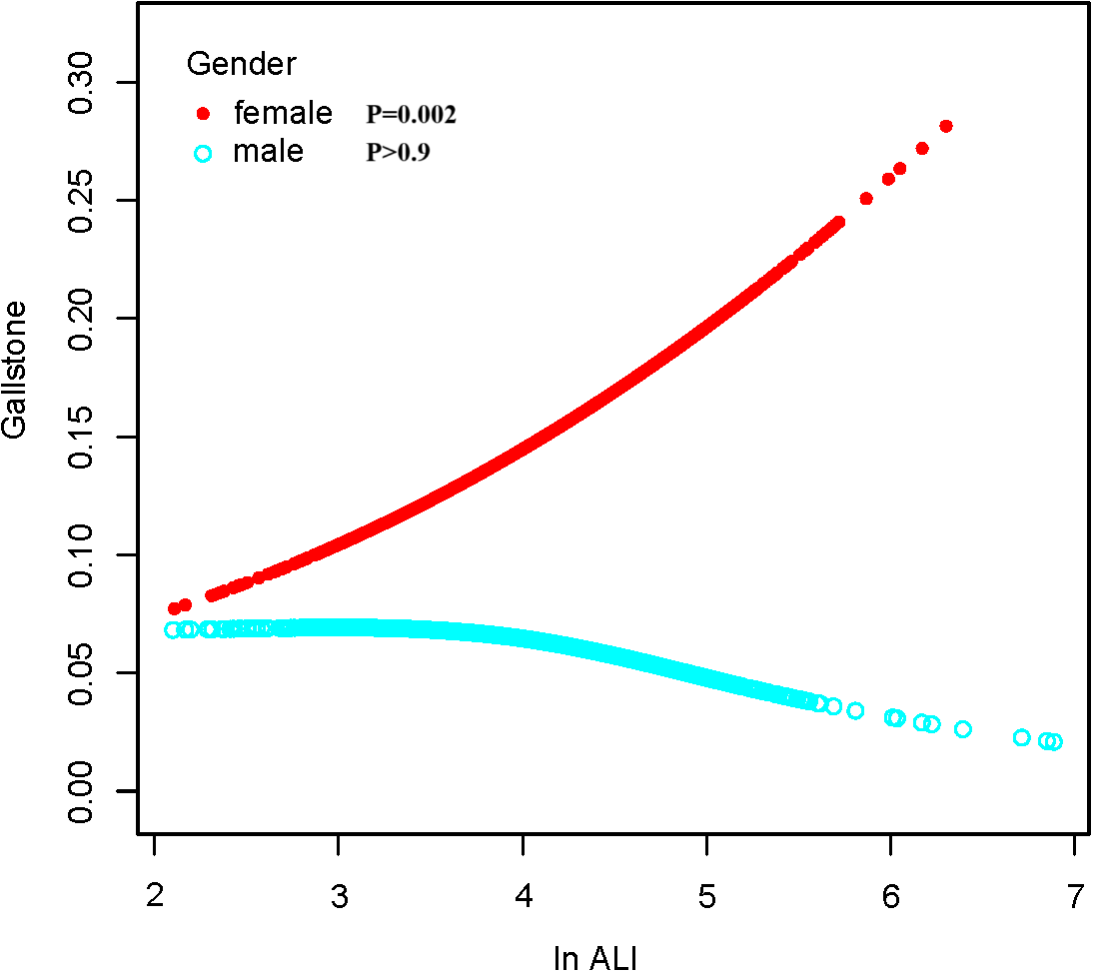
The association between ALI and gallstone stratified by sex.

To further investigate and visualize the relationship between ALI and gallstones, we utilized smoothing curve fitting to depict the association between ALI and gallstones (Fig 2). The results revealed a linear positive relationship between ALI and gallstones, consistent with the trend analysis findings presented in Table 2. When stratified by gender, the smoothing curve analysis (Fig 3) demonstrated a linear positive correlation between ALI and gallstones in females, whereas no significant correlation was observed in males. This result is consistent with the gender-stratified analysis outcomes shown in Table 3.

## Discussion

This study explored the relationship between ALI and the gallstones risk. Based on the review of data from 5,826 participants, we found that higher ALI levels were significantly linked to a higher likelihood of developing gallstones, even after accounting for multiple covariates. Trend analysis indicated a linear increase in gallstone risk with rising ALI levels, and this finding was further supported by smoothing curve fitting. Subgroup analysis revealed that the link between ALI and gallstone was stronger among females, non-diabetic individuals, individuals possessing at least a high school diploma, those who were physically inactive, and non-drinkers. Additionally, gender played a significant moderating role in the relationship between ALI and gallstones, suggesting that ALI may be a stronger predictor of gallstone risk in women.

The formation of gallstones is a complex, multifactorial process in which inflammation is considered a key pathological mechanism. Localized inflammatory responses can promote gallstone formation through various pathways [23]. First, chronic inflammation can cause thickening of the gallbladder wall and dysfunction of the biliary system, leading to impaired bile flow, which in turn allows cholesterol to precipitate more easily, thereby increasing the risk of gallstone formation [24]. Additionally, the supersaturation of cholesterol in bile is a critical factor in cholesterol stone formation. Inflammation can alter the composition of bile, particularly by disrupting the balance of cholesterol, phospholipids, and bile acids, which collectively accelerate the crystallization of cholesterol [25]. Studies have also shown that chronic inflammation in the gallbladder and bile ducts may shorten the nucleation time of cholesterol crystals (i.e., the initial stage of crystal formation in bile), further accelerating gallstone formation [26]. Neutrophils, a crucial element in innate immunity, are often elevated in bacterial infections, acute inflammation, and certain inflammatory diseases [27]. Lymphocytes, which play a crucial role in adaptive immunity, are also linked to inflammatory diseases, whether their levels are elevated or reduced [28]. Consequently, the Neutrophil-to-Lymphocyte Ratio (NLR) can reflect the body’s immune response and inflammatory state. Previous studies have indicated that the Systemic Immune-Inflammation Index (SII), calculated from platelet, neutrophil, and lymphocyte counts, serves as a marker of inflammatory status, with higher SII levels being closely associated with a higher prevalence of gallstones in individuals under 50 years of age [29]. Beyond inflammation, nutritional status also plays a significant role in gallstone formation. BMI and serum albumin levels are two key indicators of nutritional status [30]. A large cohort study by Unalp-Arida et al. demonstrated a strong link between elevated BMI and the development of gallstones [31]. Zhang et al.’s study found that obesity is associated with the occurrence of gallstones even in metabolically healthy adults [32]. Additionally, recent research indicates that visceral fat accumulation is closely linked to the risk of gallstones [33]. In our study, we similarly found that patients with a BMI greater than 30 had a higher prevalence of gallstones. This is likely because the livers of obese individuals tend to synthesize and secrete more cholesterol into bile, increasing bile cholesterol saturation and promoting stone formation [34]. Moreover, obese individuals often have poor gallbladder contractility, leading to delayed gallbladder emptying, which prolongs bile retention and further increases the risk of stone formation [35]. Additionally, obesity is closely linked to insulin resistance and metabolic syndrome, which can further elevate the risk of gallstone formation [36]. In summary, inflammation and nutritional status play crucial roles in the development of gallstones, and their interaction further amplifies this risk.

Numerous studies have confirmed the critical role of inflammation and nutritional status in the formation of gallstones. Liu et al., analyzing data from 95,319 participants, found that increased levels of high-sensitivity C-reactive protein (hs-CRP) independently contribute to the risk of new-onset gallstones in the Chinese population [37]. Another study demonstrated that the monocyte-to-high-density lipoprotein cholesterol ratio (MHR), a combined marker reflecting inflammation and oxidative stress, is positively correlated with the prevalence of gallstones [38]. Cheng et al.‘s cross-sectional study, which analyzed data from 7,334 participants, showed that a high dietary inflammatory index, linked to pro-inflammatory diets, significantly increases the risk of gallstones [39]. Furthermore, recent studies have found that various BMI-related indices effectively reflect nutritional status and are closely associated with gallstones. Zhang et al. reported that an elevated visceral adiposity index is strongly linked to the prevalence of gallstones [40], while the weight-adjusted waist index is also positively correlated with the prevalence of cholelithiasis [41]. Although numerous studies have established the close relationship between inflammation or nutritional condition and gallstones, most have focused on single factors, lacking systematic analysis of the combined effects of multiple factors. S2 Table in S1 File provides a summary of ongoing and completed clinical trials related to inflammation, nutrition, and gallstone risk. These trials highlight the growing clinical interest in exploring the combined influence of these factors, reinforcing the importance of a comprehensive approach. The ALI, composed of BMI, serum albumin levels, and NLR, can comprehensively reflect the role of inflammation and nutrition on gallstones. Our study found that higher ALI levels were strongly linked to a higher likelihood of developing gallstones.

Moreover, our analysis revealed that gender plays a notable moderating role in the relationship between ALI and gallstone risk, with the association being more pronounced in females. This occurrence could be due to the physiological and immune system differences unique to women. Hormonal fluctuations during physiological processes such as pregnancy and menopause significantly affect cholesterol metabolism and bile composition, especially post-menopause, where women have a higher cholesterol burden than men [42]. Research indicates that women are twice as likely as men to develop cholesterol gallstones, as estrogen promotes cholesterol secretion into bile via the estrogen receptor (ER) α pathway, leading to bile becoming more prone to cholesterol supersaturation, thus increasing the risk of gallstone formation [43]. Furthermore, literature suggests that women’s immune systems are generally more active than men’s, exhibiting elevated neutrophil levels and heightened sensitivity to pro-inflammatory signals [44]. Therefore, when ALI levels increase, women might be more vulnerable to nutritional status and inflammatory responses, thereby increasing the risk of gallstones. Liu et al.’s study similarly found that women are more affected by inflammatory markers such as MHR, leading to an increased risk of gallstones [38]. This finding aligns with our study’s results. In our research, we also observed that the correlation between ALI and gallstone risk was significantly enhanced in non-diabetic patients, those with a high school education or higher, those who were physically inactive, and non-drinkers. These results suggest that the impact of ALI may vary across different populations. For instance, diabetic patients have been shown to have a significant causal relationship with gallstones, with their likelihood of developing gallstones being considerably higher due to metabolic dysfunction [45]. In contrast, non-diabetic patients, with relatively stable metabolism, may be more directly impacted by changes in ALI. Similarly, individuals with higher education levels may be more focused on health management, making the association between ALI fluctuations and gallstone risk more apparent. Physically inactive individuals may experience the effects of ALI more prominently due to metabolic and gallbladder function issues [46]. Alcohol, in certain cases, is a protective factor against gallstones, likely due to its influence on cholesterol metabolism [47]. Our study found that non-drinkers exhibited a greater prevalence of gallstones than drinkers. Therefore, in non-drinkers, elevated ALI levels may more directly reflect an increased risk of gallstones. These findings suggest that ALI could serve as a valuable instrument in clinical settings for evaluating gallstone risk in specific high-risk populations. Particularly in women, non-diabetic patients, individuals with higher education levels, those who are physically inactive, and non-drinkers, early monitoring and intervention based on ALI levels could help reduce the incidence of gallstones. Future research should further explore the characteristics of these subgroups to develop more precise prevention and treatment strategies.

This study possesses several strengths. First, it leverages the large-scale NHANES database, which is nationally representative and has been weighted to ensure external validity and nationwide representativeness of the results. Second, by comprehensively adjusting for confounding factors like age, gender, race, and education level, the findings of the study become more accurate and reliable. Additionally, detailed subgroup analyses were conducted, revealing heterogeneity in the relationship between ALI and gallstone risk across different populations, thereby providing scientific evidence for personalized prevention and treatment strategies. The study also employed the innovative ALI index, which integrates multiple factors related to inflammation and nutritional status, offering a more comprehensive assessment of overall health. Lastly, trend analysis and smooth curve fitting further validated the linear relationship between ALI and gallstone risk, enhancing the robustness of the study’s findings.

This study also has some limitations that should be considered. First, since the study is based on a retrospective and cross-sectional design, we are unable to establish causality. Although we observed an association between ALI and gallstone risk, it is unclear whether an increase in ALI leads to gallstone formation or whether the presence of gallstones causes an increase in ALI. Therefore, prospective longitudinal studies are needed in the future to further confirm this causal relationship. Second, this research relies on pre-existing data from the NHANES database, which includes self-reported information. This introduces the potential for recall bias and subjective error, particularly regarding health status and lifestyle factors. Although NHANES is managed by the CDC with rigorous protocols to ensure data quality and national representativeness, these limitations are inherent to retrospective surveys. Additionally, while we controlled for multiple confounding factors, some unmeasured confounders may still influence the results. Third, this study is limited by the lack of detailed information on the types of gallstones (e.g., cholesterol stones, brown pigment stones, and black pigment stones). These types have distinct etiologies and pathogenesis, which could affect the association between ALI and gallstone risk. Unfortunately, the NHANES dataset does not provide such classification, limiting our ability to explore these potential differences. Future research incorporating datasets with detailed gallstone classifications would help further validate and refine these findings. Lastly, this study is based solely on data from the U.S. population, which may introduce geographical and cultural limitations. Differences in lifestyle, healthcare systems, and genetic backgrounds in other regions may affect the relationship between ALI and gallstone risk. Future research should validate these findings using datasets from other countries and cultural contexts to ensure broader applicability and improve generalizability.

## Conclusions

This study is the first to reveal a significant association between ALI and the risk of gallstones, particularly among women. This finding lays a critical foundation for future research and clinical applications, suggesting that incorporating ALI into routine clinical risk assessment tools could facilitate the early identification of high-risk individuals and the development of targeted prevention strategies, such as lifestyle interventions or medical monitoring, to reduce the occurrence of gallstones. Additionally, since ALI is calculated using easily accessible parameters—BMI, serum albumin, and NLR—it is both simple and cost-effective, making it suitable not only for clinical practice but also for community screening programs, thereby helping to alleviate healthcare burdens. Future research could further explore the value of ALI in personalized management and validate its predictive capability across different populations.

## Supporting information

S1 File Supplementary materials. This file includes the variance inflation factors for variables (**S1 Table**), heatmap of Spearman’s rank correlation coefficients (**S1 Fig**), summary of clinical trials related to inflammation, nutrition, and gallstone risk (S2 Table), and data processing and modeling methods (S3 Table).
(DOCX).
