# Supplementary material for: Association between advanced lung cancer inflammation index and gallstone prevalence among U.S. adults: A population-based study
Source: PLoS One. 2025 Apr 15;20(4):e0321733. doi: 10.1371/journal.pone.0321733 (PMC11999145; doi:10.1371/journal.pone.0321733)
Supplement: S1 File — This file includes the variance inflation factors for variables (S1 Table), heatmap of Spearman’s rank correlation coefficients (S1 Fig), summary of clinical trials related to inflammation, nutrition, and gallstone risk (S2 Table), and data processing and modeling methods (S3 Table). (DOCX). [file pone.0321733.s001.docx]

| Variables | GVIF | Df | GVIF^(1/(2*Df)) |
| --- | --- | --- | --- |
| ln ALI | 1.08711589 | 1 | 1.0426485 |
| Age | 1.44561368 | 1 | 1.20233676 |
| PIR | 1.31483275 | 1 | 1.14666157 |
| Gender | 1.06822853 | 1 | 1.03355142 |
| Race | 1.26079695 | 4 | 1.02939166 |
| Education level | 1.25590893 | 1 | 1.12067343 |
| Physical activity | 1.13390157 | 1 | 1.06484815 |
| Smoking status | 1.22140817 | 2 | 1.05127226 |
| Diabetes | 1.1789735 | 1 | 1.08580546 |
| Alcohol | 1.09549771 | 1 | 1.04666026 |
| Hypertension | 1.29085166 | 1 | 1.13615653 |

**S1 Table. Variance inflation factors for variables.**

ALI, advanced lung cancer inflammation index; GVIF, Generalized Variance Inflation Factor; Df, Degrees of Freedom.


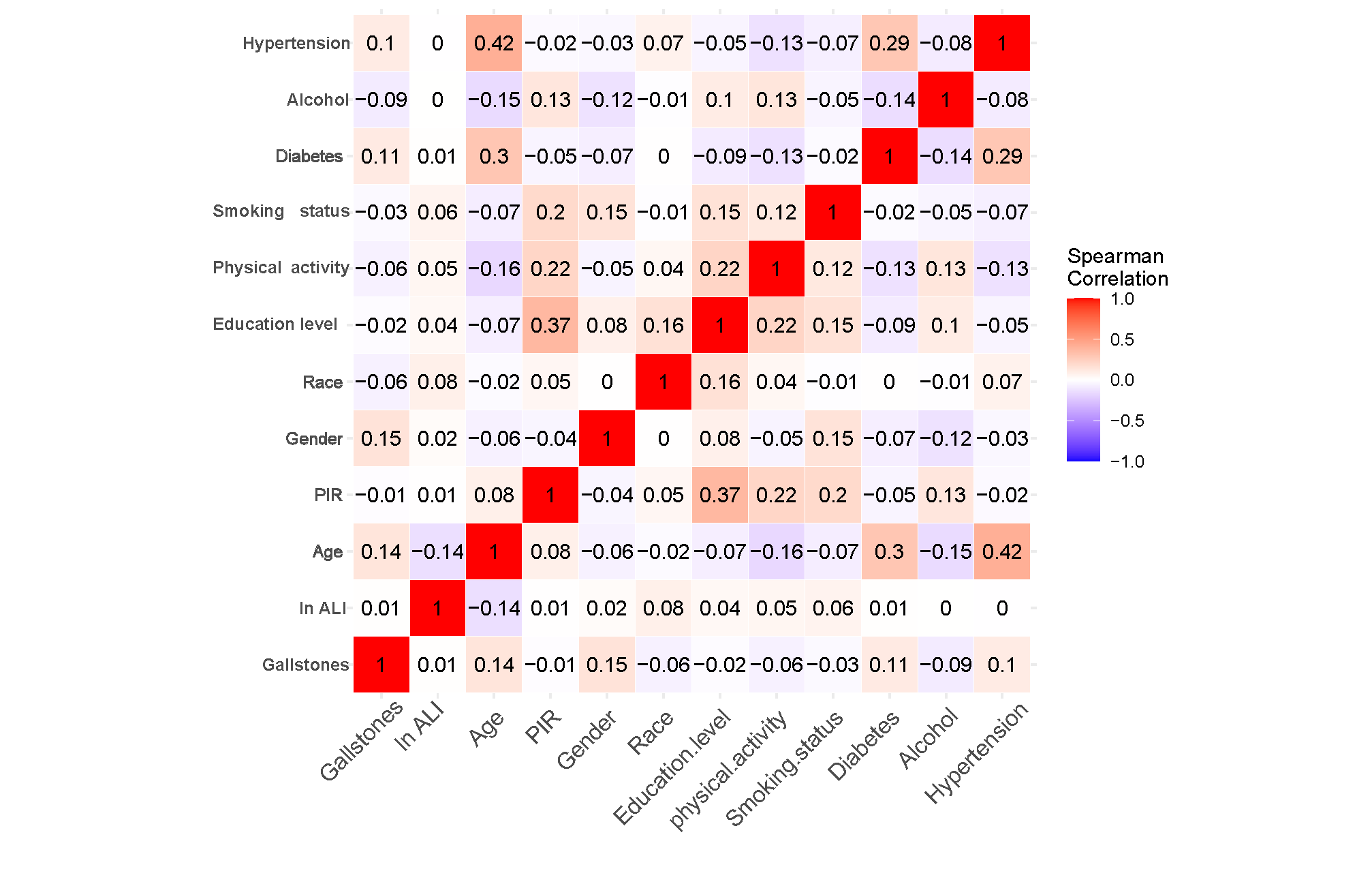


**S1 Fig.** **Heatmap of Spearman’s rank correlation coefficients.**

**S2 Table. Summary of clinical trials related to inflammation, nutrition, and gallstone risk.**

|  | Study Population | Exposure | Primary Outcome | Date | Reference |
| --- | --- | --- | --- | --- | --- |
| Inflammation and Gallstone Risk | 299 participants (China) | Circulating inflammation proteins | Risk of Gallstone Formation | 2018.11 | [10] |
|  | 150 female participants (Iran) | Dietary inflammatory index | Risk of Gallstone Formation | 2023.09 | [9] |
|  | 10779 participants (America) | Dietary inflammatory index | Risk of Gallstone Formation | 2024.05 | [8] |
|  | 2,420 participants under 50 years old (America) | systemic immune-inflammatory index | Risk of Gallstone Formation | 2024.01 | [29] |
|  | 5907 participants (America) | monocyte-to-high-density lipoprotein-cholesterol ratio | Risk of Gallstone Formation | 2024.06 | [38] |
| Nutrition and Gallstone Risk | 7409 participants (America) | visceral adiposity index | Risk of Gallstone Formation | 2023.06 | [40] |
|  | 7971 participants (America) | weight-adjusted waist circumference index | Risk of Gallstone Formation | 2023.10 | [41] |
|  | 3582 participants (America) | lipid accumulation products | Risk of Gallstone Formation | 2024.09 | [33] |
|  | 125,668 participants (China) | Metabolically healthy overweight/obesity | Risk of Gallstone Formation | 2023.03 | [32] |

**S3 Table. Data Processing and Modeling Methods**

| **Section** | **Description** |
| --- | --- |
| **1. Data Source and Study Design** | **Data Source:** NHANES data from 2017 to March 2020. **Study Design:** Cross-sectional study. **Sample Size:** 5,826 participants. |
| **2. Data Cleaning and Preprocessing** | 1.Exclusion of participants with missing variables.  2.Log-transformation of ALI (ln ALI). |
| **3. Descriptive Analysis** | 1.Calculation of weighted means and standard errors for continuous variables.  2.Calculate the counts and weighted proportions of categorical variables.  3.Group comparisons using t-tests and chi-squared tests. |
| **4. Multivariable Logistic Regression Models** | **Model 1:** No covariate adjustments.  **Model 2:** Adjusted for age, gender, and race.  **Model 3:** Further adjusted for education level, smoking status, physical activity, alcohol consumption, hypertension, diabetes, and PIR. |
| **5. Quartile Analysis and Trend Tests** | 1.Division of ALI into **quartiles (Q1-Q4)**.  2.Calculation of ORs for each quartile and conducting trend tests. |
| **6. Subgroup and Interaction Analysis** | 1.Subgroup analysis to assess the relationship between ALI and gallstone risk across variables such as gender, diabetes status, and education level.  2.Evaluate the moderating effects of variables using interaction terms. |
| **7. Smoothing Curve Fitting** | Application of **smoothing splines** to visualize the relationship between ALI and gallstones, with stratification by gender. |
| **8. Software and Tools Used** | **R Studio (4.3.3):** For statistical analysis, modeling, subgroup analysis, and interaction effect analysis.  **Empower Software (4.2):** For smoothing curve fitting. |
| **9. Example R Code** | **R Code Example:**  NHANES_design <- svydesign(data = paper.data, ids = ~SDMVPSU, strata = ~SDMVSTRA,  nest = TRUE, weights = ~WTMECPRP, survey.lonely.psu = "adjust")  m1 <- svyglm(gallstone ~ log_ali,  design = NHANES_design, family = quasibinomial)  m1 <- svyglm(gallstone ~ log_ali + Age + factor(Race) + factor(Gender), design = NHANES_design, family = quasibinomial)  m1 <- svyglm(gallstone ~ log_ali + Age + PIR + factor(Gender) + factor(Race) + factor(Education.level) + factor(Physical.activity) + factor(Smoking.status) + factor(Diabetes) + factor(Alcohol) + factor(Hypertension), design = NHANES_design, family = quasibinomial) |
